# Supplementary material for: Elevated Circulating and Placental SPINT2 Is Associated with Placental Dysfunction
Source: Int J Mol Sci. 2021 Jul 12;22(14):7467. doi: 10.3390/ijms22147467 (PMC8305184; doi:10.3390/ijms22147467)
Supplement: Supplementary file 1 [file ijms-22-07467-s001.zip › ijms-1288125-supplementary.pdf]

Supplementary Materials

Elevated circulating and placental SPINT2 is associated with placental dysfunction

Ciara N Murphy[1,2*], Susan P Walker[1,2], Teresa M MacDonald[1,2], Emerson Keenan[1,2], Natalie J Hannan[1,2], Mary E Wlodek[1,3], Jenny Myers[4], Jessica F Briffa[3], Tania Romano[5], Alexandra Roddy-Mitchell[1,2], Carole-Anne Whigham[1,2], Ping Cannon[1,2], Tuong-Vi Nguyen[1,2], Manju Kandel[1,2], Natasha Pritchard[1,2], Stephen Tong[1,2^], Tu'uhevaha J Kaitu'u-Lino[1,2^]

[1] The Department of Obstetrics and Gynaecology, Mercy Hospital for Women, The University of Melbourne, Heidelberg, 3084, Victoria, Australia.
[2] Mercy Perinatal, Mercy Hospital for Women, Heidelberg, 3084, Victoria, Australia.
[3] The Department of Anatomy and Physiology, The University of Melbourne, 3010, Victoria, Australia.
[4] University of Manchester, Manchester Academic Health Science Centre, St Mary's Hospital, Manchester, M13 OJH United Kingdom.
[5] The Department of Physiology, Anatomy and Microbiology, La Trobe University, Bundoora, 3086, Victoria, Australia.
* Correspondence: ciaram1@student.unimelb.edu.au
^ Equal contribution.

Table S1: MAViS clinical samples – FGR

Multivariate linear regression for the MAViS cohort, comparing uncomplicated pregnancies (n=175) to SGA pregnancies (with or without PE; n=84). Given the wide gestational range at sampling and underlying hypertension, multivariate linear regression with respect to the logSPINT2 values was performed to account for gestation at sampling and hypertensive status in the MAViS cohort.

Variable	Coefficient estimate	95% CI	p value
Chronic hypertension	-0.14	-0.46 to 0.19	0.40
Renal hypertension	0.23	-0.19 to 0.66	0.29
Small for gestational age[1]	0.05	-0.20 to 0.31	0.68
Gestation at sampling	0.0044	-0.0022 to 0.0110	0.19

[1] SGA used as proxy for FGR.

Table S2: MAViS clinical samples – preeclampsia

Multivariate linear regression for the MAViS cohort comparing uncomplicated pregnancies (n=175) to those complicated by PE (n=57). Given the wide gestational range at sampling and underlying hypertension, multivariate linear regression with respect to the log SPINT2 values was performed to account for gestation at sampling and hypertensive status in the MAViS cohort.

Variable	Coefficient estimate	95% CI	p value
Chronic hypertension	-0.21	-0.57 to 0.16	0.27
Renal hypertension	0.20	-0.28 to 0.68	0.42
Preeclampsia	0.20	-0.11 to 0.51	0.20
Gestation at sampling	0.0024	-0.0049 to 0.0097	0.52

Table S3: Patient characteristics of preterm (<34 weeks) placental tissue

		control n=19	FGR n=14	FGR & PE n=20	PE n=60
	**gestation at delivery** (days) mean (SEM)	211 (4.13)	219 (2.76)	204 (4.37)	209 (2.09)
	**birth weight** (g) mean (SEM)	1557 (108.6)	1040 (72.1) **	863 (80.2) ****	1251 (56.7)
**fetal sex** no. (%)	**male**	10 (52.6)	8 (57.1)	8 (40)	33 (55)
	**female**	9 (47.4)	6 (42.9)	12 (60)	27 (45)
	**maternal age** (years) mean (SEM)	30.8 (1.7)	28.9 (1.9)	29.0 (1.1)	31.2 (0.7)
	**BMI** (kg/m^2) median (IQR)	28.2 (24.8-35.1)	23.6 (18.9-29.5)	28.6 (25.0-35.5)	27.0 (24.0-36.5)
**parity** no. (%)	0	4 (21.1)	10 (71.3)	17 (85.0)	38 (63.3)
	1	10 (52.6)	2 (14.3) *	2 (10.0) ***	15 (25.0) **
	≥2	5 (26.3)	2 (14.3)	1 (5.0)	7 (11.7)
**blood pressure** median (IQR)	**systolic BP** at delivery (mmHg)	125.0 (112.0-130.0)	122.5 (116.5-133.5)	170.0 (155.0-178.8) ****	175.0 (165.0-180.0) ****
	**diastolic BP** at delivery (mmHg)	75.0 (70.0-80.0)	80.0 (70.0-85.0) *	102.5 (96.3-110.0) ****	100.0 (100.0-110.0) ****
**smoking** no. (%)	smoker	4 (21.0)	5 (35.7)	1 (5.9)	2 (3.7)
	former smoker	1 (5.3)	2 (14.3)	1 (5.9)	3 (5.6)
	non-smoker	14 (73.7)	7 (50.0)	15 (88.2)	49 (90.7)

FGR = Fetal Growth Restriction; PE = preeclampsia; BMI = body mass index, defined as weight relative to height kg/m^2; data missing for 5/19 controls, 3/20 FGR & PE samples and 9/60 PE samples. Blood pressure data missing for 1/60 PE patient. Smoking status unavailable for 3/20 FGR & PE samples and 6/60 PE samples. Kruskal-Wallis tests followed by Dunn's multiple comparisons post-hoc tests were used for comparison of non-parametric medians; normally distributed data were analysed using ordinary one-way ANOVA; categorical variables were assessed using χ-square test. *p<0.05, **p<0.01, ***p<0.001, ****p<0.0001.

Table S4: Patient characteristics of preterm (<34 week) plasma

			control n=26	FGR n=6	FGR & PE n=11	PE n=40
		**gestation at collection** (days) median (IQR)	197 (182.3-217.0)	217 (189.0-229.8)	192 (175.0-204.0)	214 (191.3-222.5)
		**birth weight** (g) median (IQR)	3458 (3180-3758)	2530 (1017-2778) *	653 (515-749) ****	1277 (846-1628) ****
**fetal sex**	no. (%)	**male**	13 (50)	1 (16.7)	4 (36.4)	18 (45)
		**female**	13 (50)	5 (83.3)	7 (63.6)	22 (55)
		**maternal age** (years) median (IQR)	32.0 (28.5-34.5)	31.5 (29.0-36.8)	32.0 (30.0-38.0)	32.0 (28.3-34.0)
		**BMI** (kg/m^2) median (IQR)	24.2 (21.1-28.2)	27.1 (22.9-37.4)	32.0 (27.1-39.0) **	28.6 (25.0-35.2) **
**parity**	no. (%)	0	10 (38.5)	4 (66.7)	7 (63.6)	27 (67.5)
		1	11 (42.3)	1 (16.7)	2 (18.2)	9 (22.5)
		≥2	5 (19.2)	1 (16.7)	2 (18.2)	4 (10.0)
**blood pressure**	median (IQR)	**systolic BP** at delivery (mmHg)	120 (112.5-128.8)	115 (107.5-121.3)	170 (170.0-180.0) ****	175 (165.0-180.0) ****
		**diastolic BP** at delivery (mmHg)	75 (70.0-80.0)	73 (67.5-80.5)	100 (90.0-110.0) ****	102 (100.0-110.0) ****

Maternal age not available for 1/26 controls. BMI = body mass index, defined as weight relative to height kg/m^2; data missing for 4/40 PE samples. Blood pressure data missing for 2/26 controls. Kruskal-Wallis tests followed by Dunn's multiple comparisons post-hoc tests were used for comparison of non-parametric medians; normally distributed data were analysed using ordinary one-way ANOVA; categorical variables were assessed using χ-square test. *p<0.05, **p<0.01, ***p<0.001, ****p<0.0001.

Table S5: Patient characteristics of FLAG2 plasma (delivery at term)

		control n=152	SGA n=75
	**gestation at collection** (days) median (IQR)	272 (269-273)	270 (264-273) ***
	**birth weight** (g) median (IQR)	3510 (3183-3798)	2660 (2510-2830) ****
**fetal sex** no. (%)	**male**	70 (46.1)	41 (54.7)
	**female**	82 (53.9)	34 (45.3)
	**BMI** (kg/m^2) median (IQR)	26.6 (23.4-32.1)	25.0 (21.9-30.1) *
**parity** no. (%)	0	34 (22.4)	13 (17.3)
	1	82 (53.9)	45 (60.0)
	≥2	36 (23.7)	17 (22.7)

SGA was defined as a birthweight <10th centile (GROW bulk centile), and control was defined as BWC >10th. BMI = body mass index, defined as weight relative to height kg/m^2; data missing for 5/152 controls and 2/75 SGA samples. Mann-Whitney U tests were used for comparison of medians and categorical variables were assessed using χ-square test. *p<0.05, **p<0.01, ***p<0.001, ****p<0.0001.

Table S6: Patient characteristics of BUMPS plasma (36 weeks)

		**control** n=182	**FGR** n=121		**FGR & PE** n=7	**PE** n=16	
	**gestation at collection** (weeks) median (IQR)	36.1 (35.7-36.6)	36.1 (35.7-36.4)		36.3 (36.1-36.6)	36.2 (35.5-36.7)	
	**birthweight** (grams) median (IQR)	3490 (3185-3820)	2800 (2578-2970) ****		2542 (2420-2660) ****	3315 (3128-3555)	
	**crown-heel length** (cm) median (IQR)	51 (50-52)	49 (47-50) ****		48 (46-51) **	52 (48-53)	
**fetal sex** no. (%)	**male**	91 (50)	52 (44)		3 (43)	7 (44)	
	**female**	91 (50)	66 (56)		4 (57)	9 (56)	
	**maternal age** (years) median (IQR)	32.0 (29.8-35.0)	34.0 (30.3-36.8)		36.0 (32.0-38.0)	34.0 (30.3-36.7)	
	**BMI** (kg/m^2) median (IQR)	24.6 (22.3-28.1)	25.7 (22.2-30.1)		25.8 (23.2-30.7)	28.6 (24.8-30.6)	
**parity** no. (%)	0	89 (48.9)	79 (65.3)	*	5 (71.4)	14 (87.5)	*
	1	72 (39.6)	34 (28.1)		2 (28.6)	2 (12.5)	
	≥2	21 (11.5)	8 (6.6)		0 (0)	0 (0)	
**smoking** no. (%)	non-smoker	170 (93.4)	102 (87.2)		6 (85.7)	15 (93.8)	
	former smoker[1]	6 (3.3)	10 (8.5)		1 (14.3)	0 (0)	
	smoker	6 (3.3)	5 (4.3)		0 (0)	1 (6.2)	
**GDM** no. (%)	none	160 (88.9)	110 (91.7)		6 (85.7)	11 (68.8)	**
	diet-controlled	14 (7.8)	8 (6.7)		1 (14.3)	5 (31.2)	
	insulin-controlled	6 (3.3)	2 (1.6)		0 (0)	0 (0)	

Fetal sex unavailable for 3/121 FGR samples; BMI = body mass index, defined as weight relative to height kg/m^2; data missing for 2/182 control and 3/121 FGR samples; [1] former smokers were women who quit during this pregnancy, smoking status unavailable for 4/121 FGR samples; GDM = gestational diabetes mellitus, data missing for 2/182 control and 1/121 FGR samples. Kruskal-Wallis tests followed by Dunn's multiple comparisons post-hoc tests were used for comparison of non-parametric medians; normally distributed data were analysed using ordinary one-way ANOVA; categorical variables were assessed using χ-square test. *p<0.05, **p<0.01.

Table S7: Patient characteristics of MAViS plasma (24–34-week)

		control n=179	FGR n=58	PE n=57
	**gestation at collection** (days) median (IQR)	191 (184-214)	188 (183-211)	186 (182-203) *
	**birthweight centile** median (IQR)	45.8 (26.4-66.4)	5.60 (3.36-8.12) ****	12.5 (2.88-46.1) ****
	**maternal age** (years) median (IQR)	35.0 (32.0-38.0)	34.5 (30.0-38.3)	34.0 (30.5-36.5)
	**BMI** (kg/m^2) median (IQR)	30 (25-34)	29 (25-34)	30 (25-34)
**blood pressure** median (IQR)	**systolic BP** at delivery (mmHg)	128 (120-138)	127 (120-140)	129 (120-140)
	**diastolic BP** at delivery (mmHg)	80 (72-88)	80 (72-88)	80 (74-88)
**underlying hypertensive status** no. (%)	chronic	118 (65.9)	41 (70.7)	44 (77.2)
	renal	31 (17.3)	5 (8.6)	4 (7.0)
	none	30 (16.8)	12 (20.7)	9 (15.8)
**ethnicity** no. (%)	Asian	26 (14.8)	6 (10.5)	8 (14.0)
	black	43 (24.6)	17 (29.8)	23 (40.4)
	other	18 (10.3)	9 (15.8)	5 (8.8)
	white	88 (50.3)	25 (43.9)	21 (36.8)

BMI = body mass index, defined as weight relative to height kg/m^2; ethnicity data missing for 4/179 controls and 1/58 FGR cases. One way ANOVA (Kruskal-Wallis) tests followed by Dunn's multiple comparisons post-hoc tests were used for comparison of non-parametric medians; categorical variables were assessed using χ-square test. *p<0.05, ****p<0.0001.

Table S8: TaqMan Gene Expression Primers

The PCR primers used for all genes were the FAM-labelled, Applied Biosystems™ TaqMan™ Gene Expression Assays (Thermo Fisher Scientific; Waltham, MA,USA), as below:

	Gene(s) of Interest	Housekeeper Gene(s)*
**human placenta**	*Spint2*<br>(Hs01070442_m1 SPINT2, human)	*Cyc1*<br>(H200357717_m1 CYC1, human)<br>*Top1*<br>(Hs00243257_m1 TOP1, human)
**primary trophoblasts**		*Cyc1*<br>(H200357717_m1 CYC1, human)
**hTSCs**		*Ywhaz*<br>(Hs01122454_m1 YWHAZ, human)
**rat placenta**	*rSpint2*<br>(Rn01457943_m1 SPINT2, rat)	*β-Actin*<br>(Rn00667869_m1, β-ACTIN, rat)

*where more than one housekeeper gene is listed, average was used
